# Meta-analysis of Nicorandil effectiveness on myocardial protection after percutaneous coronary intervention

**DOI:** 10.1186/s12872-019-1071-x

**Published:** 2019-06-14

**Authors:** Xiao-Tao Zhao, Chun-Fei Zhang, Qing-Jie Liu

**Affiliations:** 1 Department of Cardiology, Beijing Chao-Yang Hospital, Captial Medical University, 8 Gongti Nanlu Road, Chaoyang District, Beijing, 100020 NO China; 2 Department of Internal Medicine, General Political Department Hospital of Chinese PLA, Beijing, China; 3 National Institute for Radiological Protection, Chinese Center for Disease Control and Prevention, Chinese Center for Medical Response to Radiation Emergency, No. 2 Xinkang Street, Deshengmenwai, Beijing, 100088 China

**Keywords:** Nicorandil, Percutaneous coronary intervention, Coronary artery disease, Acute myocardial infarction, Angina, Heart function, Cardiovascular events, Meta-analysis

## Abstract

**Background:**

Using the current meta-analysis as well as systematic review, to determine the curative effect of Nicorandil in comparison of no Nicorandil after elective percutaneous coronary intervention(PCI) on patients.

**Methods:**

Published literatures were identified via a computerized literature search of CENTRAL, PubMed, Cochrane, Embase Databases of Systematic Reviews. A set of randomized trials evaluating Nicorandil in comparison of no Nicorandil administered following PCI in patients harboring coronary artery disease were included. Outcomes were revealed based on the following parameters: peak creatine kinase-MB (CK-MB) value, left ventricular ejection fraction (LVEF), peak troponin I (cTnI), and major adverse cardiovascular events (MACEs) per randomized patients.

**Results:**

We included a total of 14 RCTs involving 1864 subjects in the present review. According to this meta-analysis, LVEF was significantly improved in Nicorandil group; the peak CK-MB level and the incidence of adverse cardiovascular events were remarkably lower in Nicorandil group. Nicorandil and no Nicorandil administered group appeared to be equivalent with regards to cTnI.

**Conclusions:**

Nicorandil is effective for patients undergoing elective PCI with coronary artery disease in terms of reducing the incidence of adverse cardiovascular events as well as improving heart function. Nicorandil may exert potential role as a valid and adjunctive therapy accompanied with PCI.

## Background

Coronary artery disease, widely acknowledged as one of the most common causes of heart disease, represent a serious health burden under the background of healthcare and socio-economic policy. Disability, morbidity, and mortality caused by CHD have been increasing year by year, with a prevalence of 10% of disability and 30% of all-cause mortality [1]. Percutaneous coronary intervention (PCI) has been known as one of the most commonly used techniques in treating coronary artery disease. In 1964, Dotter et al. firstly proposed the concept of percutaneous transluminal coronary angioplasty (PTCA); and the world’s first case of PTCA treatment of coronary artery disease conducted by Gruntzig et al. [2] in 1977 has led to significant progress of the technology and has become the most widely used intervention therapy in treating coronary artery disease. However, potential shortcoming such as myocardial injury caused by PCI has become a major concern. Myocardial injury following PCI may attribute to distal microvascular embolization, vascular endothelial injury, coronary artery spasm, surgical procedures that block blood vessels, lateral occlusion caused by plaque displacement, reperfusion injury and tissue damage [3–5]. Several drugs were tested for the prevention or alleviation of such injuries, but beneficial outcomes have not been gained [6–8].

Nicorandil is a non-selective adenosine-sensitive potassium channel opener that allows the vascular smooth muscle to relax, effectively dilate the microvascular and to improve myocardial perfusion [9, 10]. Recent basic and clinical studies revealed its protective effects for myocardial injury [11, 12]. The purpose of the current study was to provide a systematic review of Nicorandil in terms of its cardio-protective effects. The aim was to synthesize the available evidence and compare data associated with the effectiveness of Nicorandil in this objective and quantitative analysis.

## Methods

### Literature search

A thorough electronic literature search was performed involving randomized controlled trials (RCTs) published in databases such as Cochrane Central Register of Clinical Trials (CENTRAL), PubMed (1966–July 2017), Cochrane Database of Systematic Reviews (Issue 1, 2017), and Embase (1980–July 2017). A literature search was conducted with the use of the following keywords: ‘coronary heart disease’, ‘cardiovascular disease’, ‘angina pectoris’, ‘myocardial infarction’, ‘CHD’, ‘Nicorandil’, ‘PCI’, ‘percutaneous coronary intervention’, and ‘PTCA’. In order to further maximize the literature data, we contacted the author through letters for test reports due to the lack of information or unknown details from several literature and meta-analysis. Additionally, we retrieved the bibliographies of the review articles as well as retrieved trials. The study was adhered to PRISMA guidelines.

### Study selection

In order to be included in the current review, the following inclusion criteria should be met: 1) patients diagnosed with established coronary artery disease or acute myocardial infarction undergoing elective PCI; 2) comparisons of Nicorandil versus placebo or no Nicorandil administered treatment; 3) the following one or more outcome measures should be reported: peak creatine kinase-MB (CK-MB) value, Left ventricular ejection fraction (LVEF), peak troponin I (cTnI), major adverse cardiovascular events (MACEs) included i) all-cause mortality; ii) new MI; iii) any revascularization; iiii) re-hospitalization rate; 4) the publications were only available in English; 5) randomized controlled trials (RCTs). We considered the following studies as excluded ones: 1) studies lack of information of both PCI and Nicorandil; 2) incomplete or incorrect data, or lack of outcome; 3) observational studies or case reports; 4) repeated publications.

A review topic (or topics) was designed to include trials that were identified from the searching results of above-mentioned features. We utilized the Thomson Research Software (EndNote X4) for the accuracy evaluation of extracted data from review. We provided original reports for further details in case of unclear information. “excluded (reason)”, “pending”, “Included” were indicated into the “notes” column. And authors would retrace “pending” reports from the references.

### Quality assessment

We utilized Cochrane handbook for Systematic Reviews of Interventions 5.1.0, which was recommended by Cochrane Collaboration, for the assessment of study quality. The items for evaluation mainly included the following seven aspects: allocation concealment; random sequence generation; incomplete outcome data; blinding of participants and provider; selective reporting and other bias; and blinding of outcome assessment. “High risk”, “low risk”, and “unclear risk” were defined respectively as the evaluation of each document that was in accordance with the above seven items.

### Data extraction

The first authors, the years of publication were extracted as general information. Parameters, such as object characteristics, follow-up duration, treatment types, and outcome measures were utilized to analyze the study. Two authors carried out the process of quality assessment, literature selection, as well as data extraction. Any arising difference was resolved by discussion with the help of a third reviewer.

### Data synthesis and analyses

We conducted the statistical analyses on the basis of the Review Manager Software (RevMan5.3) offered by the Cochrane Collaboration. Binary classification data were presented as the risk differences (RD) and its 95% CI. For continuous outcomes, we assessed the mean difference (MD) as well as its 95% CI for the result of meta-analysis. Additionally, we calculated the standardized MD (SMD) in case of the need of different scales.

Chi-squared and I^2^ tests were used to assess the heterogeneity. We selected consistency model to fit the estimated effects. When there was no significant heterogeneity (*p* > 0.1, I^2^ ≤ 50%), we assumed the fixed-effects model would be met. And if statistical heterogeneity was identified (*p* ≤ 0.1, I^2^ > 50%), we detected subgroup analyses to find the sources of heterogeneity on the basis of interventions. In addition, we used combined random-effects model due to fail to identify the sources.

## Results

### Search results

**Fig. 1:**
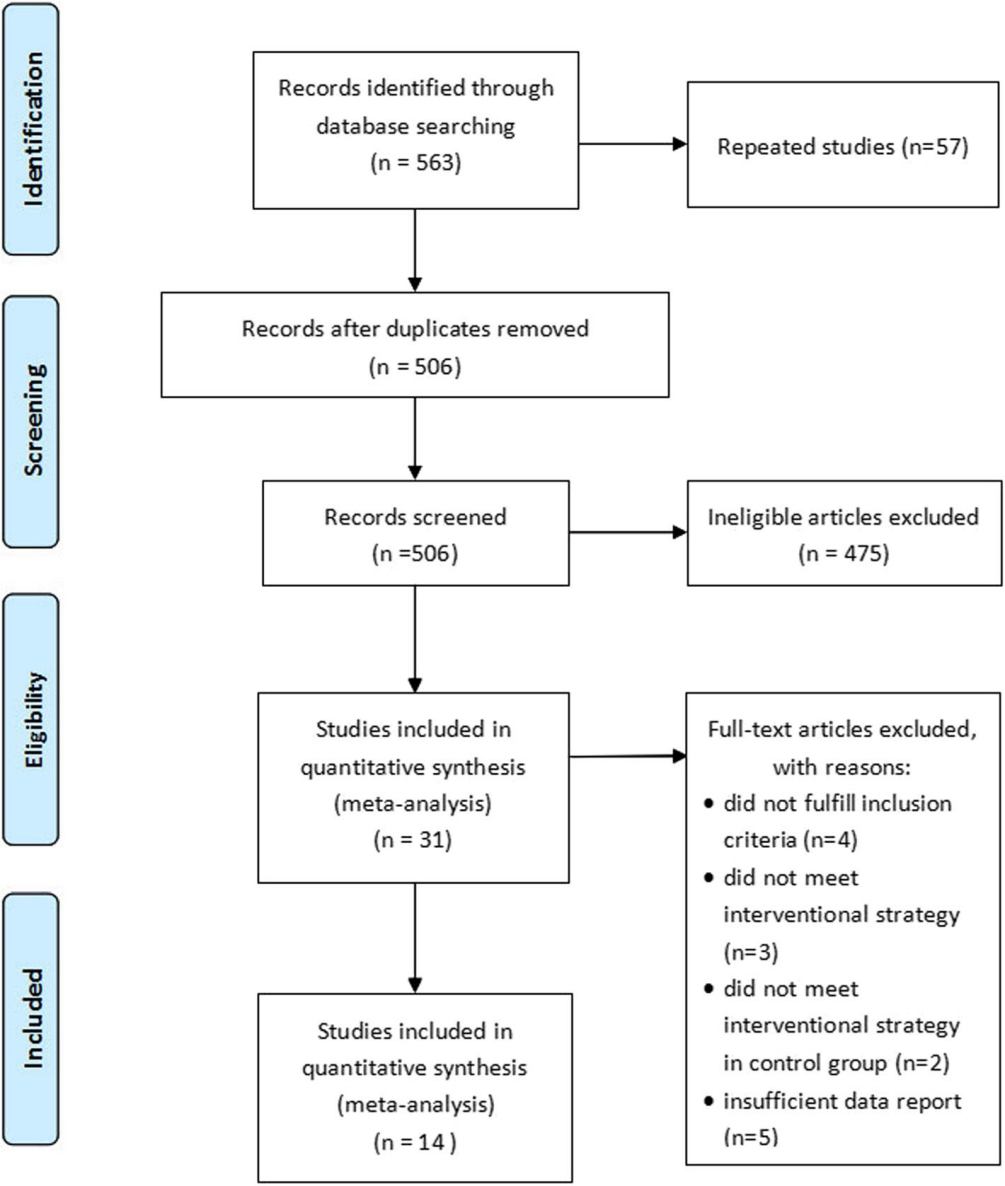
Flow diagram of search strategy of study

The abovementioned search identified 563 articles. After screening duplicates, 506 studies were kept and then 475 articles were excluded due to irrelevant citation, leaving 31 articles for further evaluation. During full-text screening, 14 articles were excluded. Finally, we included 14 RCTs published from 1999 and 2017 in the review (Fig. 1).

### Quality assessment

**Fig. 2:**
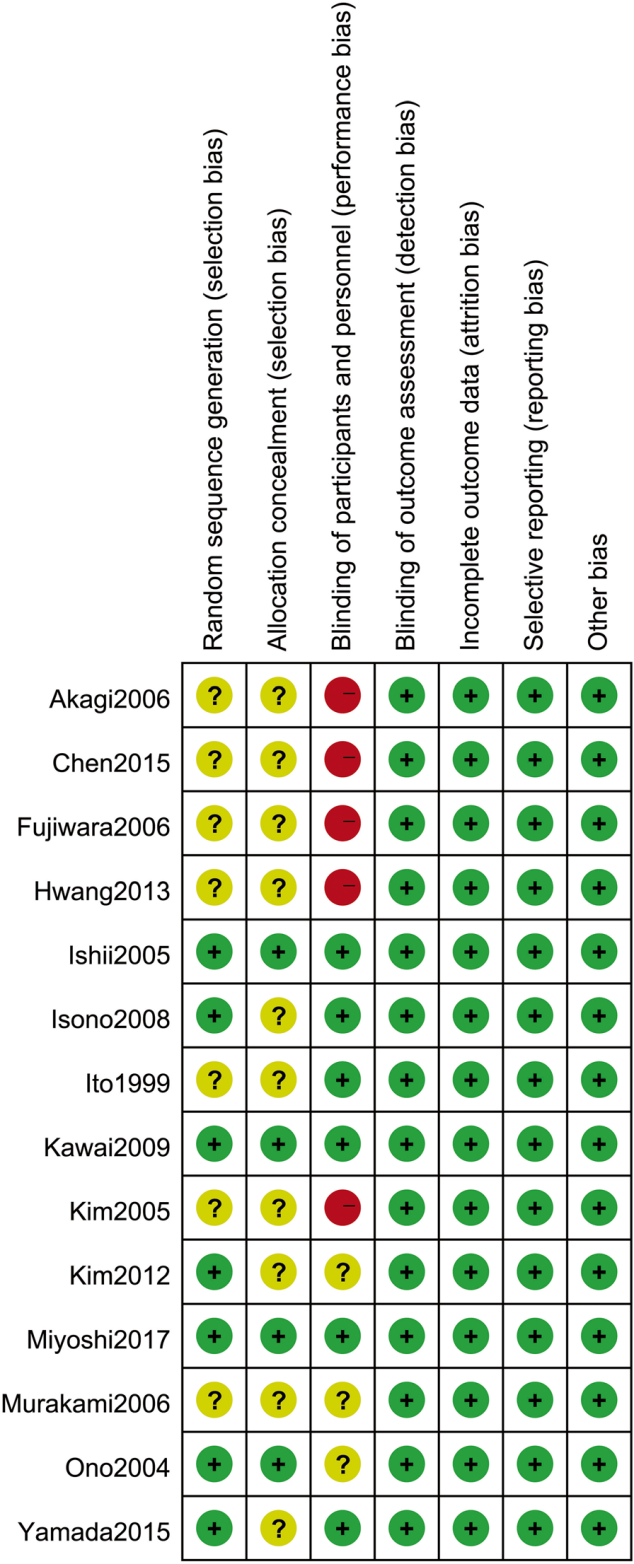
Methodological quality assessment for each included study

The overall results of quality evaluation were presented in Fig. 2 from included studies. Single-blind trials and double-blind trails were applied in 2 trials [13, 14] and 4 trials [15–18], respectively. 7 trials [13–16, 18–20] in terms of randomization were used with random number tables, computer-generated random sequence, and block randomization, while no description about the process of randomization was mentioned from other studies. Patients were distributed to 3 trials [15, 18, 20] by sealed envelope and 1 trial [13] by secure website. Comparable baselines exist in all trials. And analysis of blinded data was independent from all trials of studies. No incomplete report nor selective report were observed. Generally, the studies with moderate quality were included in this meta-analysis.

### Study characteristics

Table 1 summarized the characteristics of included studies. In total, 1864 patients (randomized population, 913 and 951 respectively in Nicorandil and control group) receiving PCI were involved in the current analysis. Patients had an average age that ranged from 57.6 to 71 years, with follow-up duration varied from 1 month to 5 years and a sample size that ranged from 20 to 408. Characteristics of experimental interventions as well as control interventions were different across the studies. Additionally, several differences were found or observed among the studies with regard of Nicorandil interventions: the routes of administration of Nicorandil, such as intravenous, intracoronary and oral routes; the administration of Nicorandil before or after PCI; the dosage of Nicorandil varied. Most of the included studies used intravenous drip infusion (4 mg/hour) before PCI for 48 h, 2 mg intracoronary and oral 15 mg/day after PCI as intervention program.

**Table 1:** Characteristics of included studies

Author, year	Samplesize, gender M/F	Mean age, years (SD)	Study population	NIC interventions	Control interventions	Outcome measures	Follow-up
Akagi, 2006 [21]	NIC: 10,6/4; 10,5/5 Control: 10,9/1	68 (9) 63 (15) 61(9)	Patients with AMI	IV drip infusion (4 mg/hr) before PCI for 48 h, 2 mg IC, and/not oral 15 mg/dayafter PCI	No NIC administered	LVEF	3 months
Chen, 2015 [22]	NIC: 26,18/8 Control: 26,19/7	57.6 (4.7) 59.8 (4.8)	Patients with AMI	IC2 mg before PCI	No NIC administered	LVEF; CK-MB; cTnI; MACEs	1 months
Fujiwara 2006 [23]	NIC: 31,25/6 Control: 31,25/6	62 (2) 62 (2)	Patients with AMI	4 mg IV before PCI, IV drip infusion (8 mg/hr) for 24 h after PCI	No NIC administered	LVEF	6 months
Hwang, 2013 [25]	NIC: 41,20/21 Control: 40,25/15	66.2 (9) 65.3 (10)	Patients with angina	IC 4 mg before PCI	No NIC administered	CK-MB; cTnI	NR
Ishii, 2005 [15]	NIC: 185,144/41 Control: 183,154/29	63 (9.4) 64 (10.1)	Patients with MI	12 mg IV over 20–30 min before reperfusion with PCI	Placebo	MACEs	5 years
Isono, 2008 [16]	NIC: 23,19/4 Control: 26,21/5	66.3 (7.9) 66.5 (9.4)	Patients with CHD	4 mg IV before PCI, IV drip infusion (6 mg/hr) for 24 h,oral15 mg/day3–6 months after PCI	No NIC administered	LVEF; CK-MB; cTnI	3 months
Ito, 1999 [17]	NIC: 40,32/8 Control: 41,32/9	60 (10) 60 (10)	Patients with first anterior AMI	4 mg IV before PCI, IV drip infusion (6 mg/hr) for 24 h,oral15 mg/day until the discharge (a mean of 28 days) after PCI	No NIC administered	LVEF; MACEs	6 months
Kawai, 2009 [18]	NIC: 206,161/45 Control: 202,149/53	69 (11.1) 71 (11.5)	Patients with/without acute coronary syndrome	6 mg IV before PCI	No NIC administered	CK-MB;MACEs	12 months
Kim, 2005 [24]	NIC: 42,27/15 Control: 54,32/22	60.4 (11.7) 61.7 (8.2)	Patients with unstable angina	4 mg IV before PCI, IV drip infusion (6 mg/hr) for 48 h,oral15 mg/day6 months after PCI	Isosorbide dinitrate	LVEF; MACEs	6 months
Kim, 2012 [19]	NIC: 54,36/8 Control: 55,46/9	65.5 (7.4) 63.2 (9.2)	Patients with stable or unstable angina	4 mg ICin 5 min before PCI	No NIC administered	MACEs	6 months
Miyoshi, 2017 [13]	NIC: 129,99/30 Control: 133,102/31	70.0 (9.2) 70.3 (10.1)	Patients with stable coronary artery disease	4 mg IV for 5 min at least 1 h before PCI, IV drip infusion (6 mg/h) for at least 8 h after PCI	No NIC administered	MACEs	8 months
Murakami, 2006 [26]	NIC: 91,75/16 Control: 101,81/20	65.0 (9.7) 66.1 (10.3)	Patients with CHD	IV drip infusion (2 mg/h) for 6 h after PCI	Placebo	CK-MB;cTnI	3 months- 3.1 years
Ono, 2004 [20]	NIC: 33,22/11 Control: 25,16/9	64 (13) 66 (12)	Patients with AMI	4 mg IV before PCI, IV drip infusion (8 mg/hr) for 24 h after PCI	No NIC administered	LVEF; MACEs	6 months
Yamada, 2015 [14]	NIC: 28,23/5 Control: 24,22/2	67 (10) 65 (12)	Patients with AMI	2 mg IC before PCI, IV drip infusion (2 mg/hr) for 4 days after PCI	Nitrate	LVEF	NR

*NIC* Nicorandil, *IC* intracoronary, *IV* intravenous, *CHD* coronary heart disease, *AMI* Acute myocardial infarction, *LVEF* Left ventricular ejection fraction, *CK-MB* creatine kinase-MB, *cTnI* troponin I, *MACEs* major adverse cardiovascular events,*NR* Not reported

### Main findings and synthesis of results

#### LVEF

**Fig. 3:**
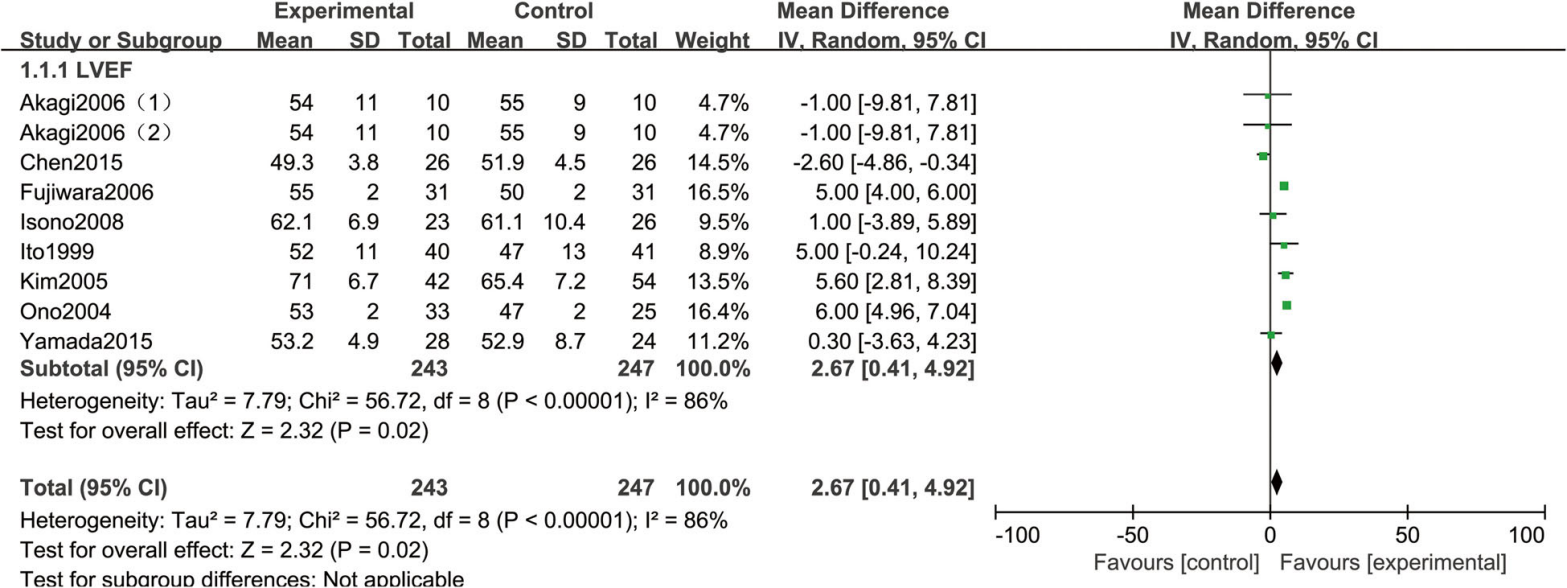
Comparison of the cardiac function between Nicorandil group and no Nicorandil group

Eight RCTs [14, 16, 17, 20–24] reported 243 and 247 patients respectively who received PCI in Nicorandil and control group. Statistical heterogeneity was observed among meta-analysis showed between the two studies (*P* < 0.00001, I^2^ = 86%), with the use of random effect model for merging, showing there was significant statistical difference of LVEF when comparing the two groups (MD = 2.67, 95% CI (0.41, 4.92), *P* = 0.02), as shown in Fig. 3

#### Peak CK-MB value and peak cTnI value

**Fig. 4:**
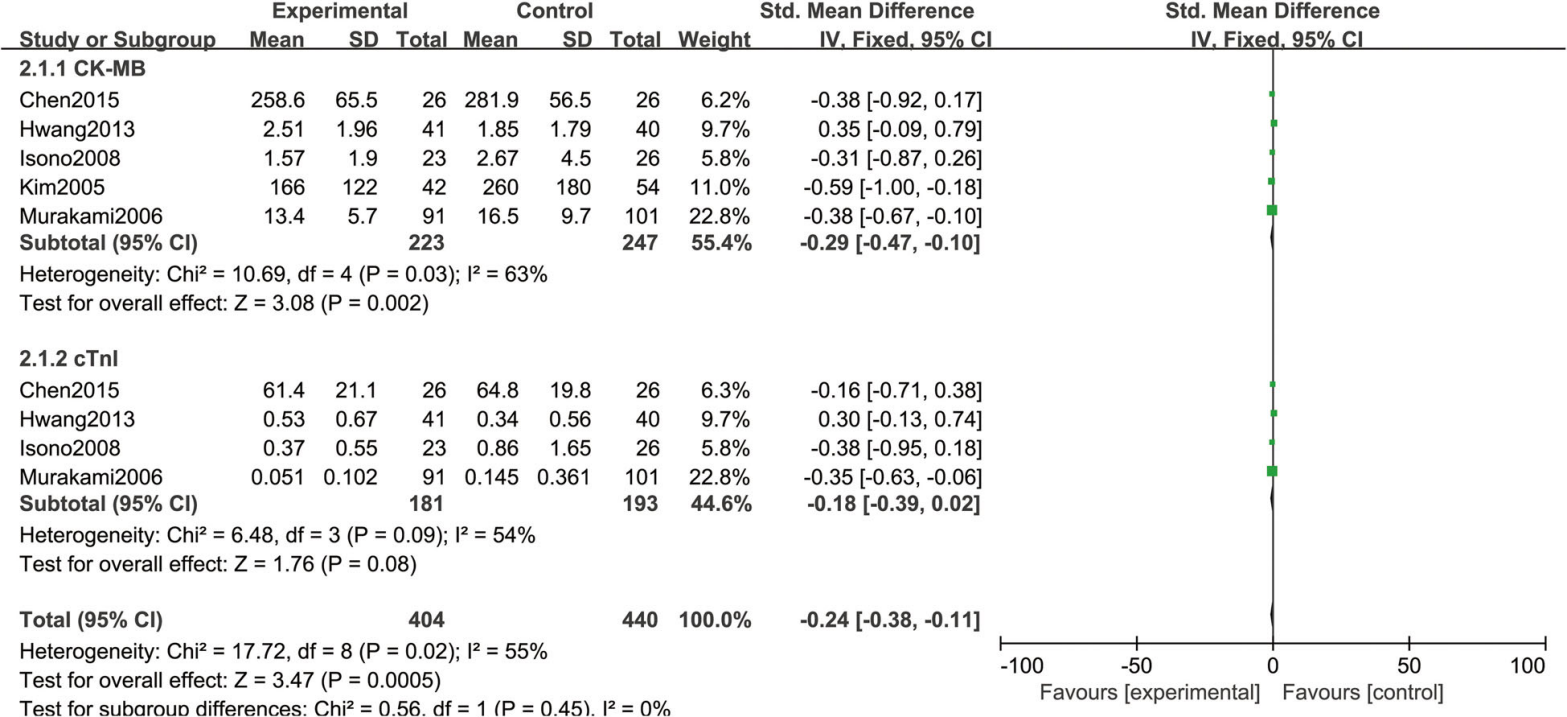
Comparison of myocardial injury indexes between Nicorandil group and no Nicorandil group

The peak CK-MB value was evaluated in 470 patients from 5 RCTs [16, 22, 24–26]. The peak cTnI value was evaluated in 374 patients from 4 RCTs [16, 22, 25, 26]. The result showed there was significant statistical difference of peak CK-MB value between Nicorandil group and control group (SMD = − 0.29, 95% CI (− 0.47, − 0.10), *P* = 0.002). Nevertheless, no significant statistical difference was found in terms of peak cTnI value (SMD = − 0.18, 95% CI (− 0.39, 0.02), *P* = 0.08), as shown in Fig. 4.

#### Major adverse cardiovascular events

**Fig. 5:**
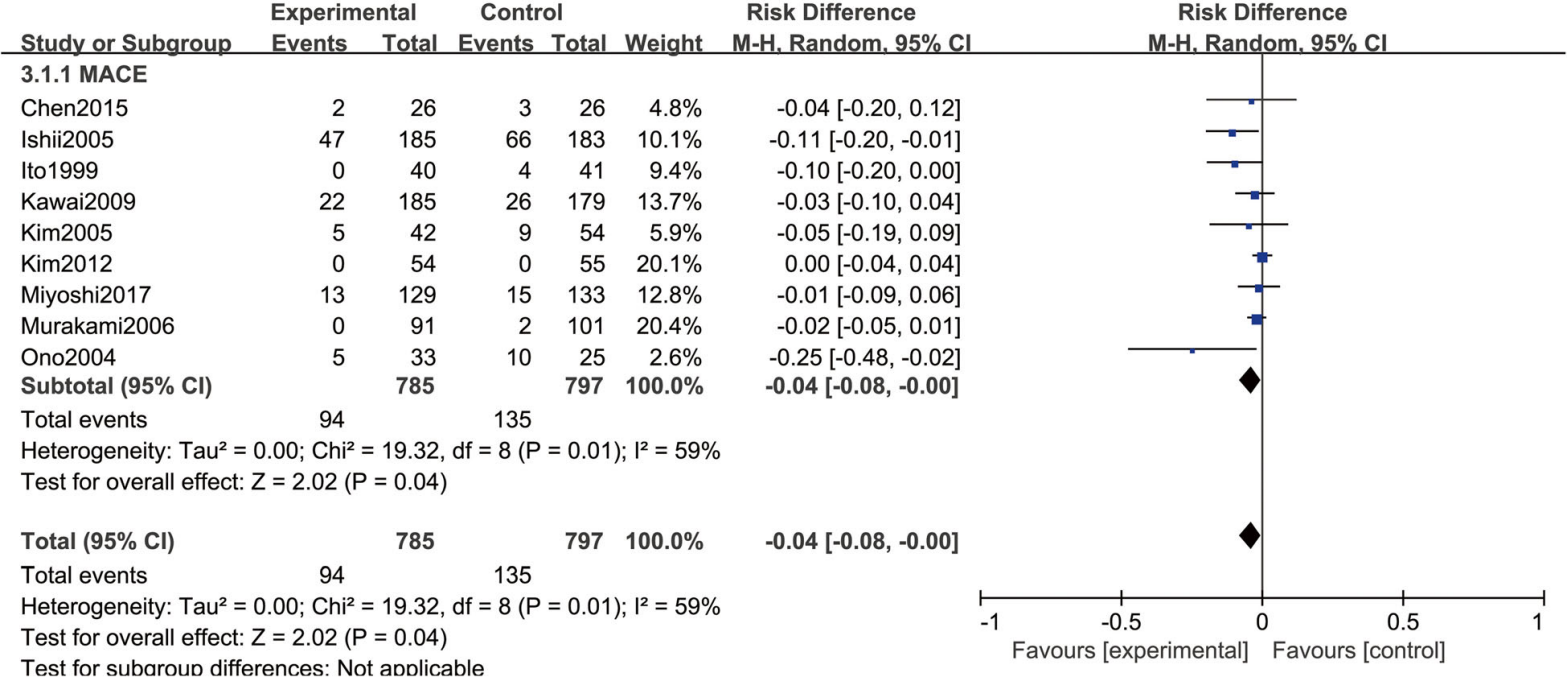
Comparison of major adverse cardiovascular events between Nicorandil group and no Nicorandil group

9 RCTs [13–15, 17–20, 22, 24, 26] revealed 785 and 797 patients respectively who received PCI in Nicorandil and control group, with 94 and 135 patients with major adverse cardiovascular events. Statistical heterogeneity was identified among the present meta-analysis comparing the studies (*P* = 0.01, I^2^ = 59%), with the application of random effect model for merging, showing there was significant statistical difference with regard to MACEs rate when comparing two groups (RD = − 0.04, 95% CI (− 0.08, − 0.00), *P* = 0.04), as shown in Fig. 5

.

## Discussion

PCI refers to the catheter through a variety of ways to expand the narrow coronary artery, with an attempt to achieve the lifting of the narrow, improve the treatment of myocardial blood supply. As a valid and alternative approach for patients harboring coronary artery anomalies, it can significantly reduce the mortality rate. However, CK-MB, cTnI and other manifestations of myocardial injury is inevitable, leading to poor prognosis. The aggregated results from the present meta-analysis showed that the level of CK-MB in patients from Nicorandil group were associated with lower trend as compared with those in the control group. In addition, 8 RCTs included in the meta-analysis showed that the left ventricular ejection function of the patients in Nicorandil group was significantly better than that in control group, and also demonstrated that the patients in Nicorandil group had stronger myocardial contractility. It is pivotal to note that the long-term prognosis such as patient mortality, re-hospitalization rate and so on were significantly lower in patients given Nicorandil than in controls. Our meta-analysis failed to show Nicorandil associated with statistically reduced cTnI, but Yang [27] reported that large doses of Nicorandil were associated with a lower incidence of serum cTnI than normal upper limit 3 times compared with low-dose Nicorandil and control groups, and the protective effect of Nicorandil on the myocardial injury was improved to some extent. Therefore, more well designed studies are needed to confirm the effect of Nicorandil in the setting of PCI.

According to several previous studies [28–30], apart from its cardioprotective effect, Nicorandil improves microvascular dysfunction. Moreover, it can be administered intravenously as an intracoronary infusion or before starting PCI. Confirmation has been gained in terms of the effectiveness of Nicorandil on stable angina in a large clinical randomized controlled trial of IONA [31] wherein there included a positive exercise test already on the optimal antianginal drug therapy or with patients harboring established coronary heart disease (previous MI, CABG). Patients in Nicorandil group (*N* = 2565) versus placebo(*N* = 2561) was significantly reduced in the incidence of adverse cardiac events, but there was no significant difference with regard to the mortality between the two groups (4.3% Vs. 5.0%, HR = 0.85, *P* = 0.222), which may be related to short follow-up time. It was consistent with subgroup analyses in a Japanese retrospective study (JCAD) [32], the study reported that Nicorandil in treating patients with coronary artery disease can significantly reduce cardiac death, fatal myocardial infarction, cerebrovascular death, congestive heart failure, re-admission rate and other end-point indicators.

The mechanisms of Nicorandil in reduced myocardium injury as well as improved heart function are suggested to attribute to several factors. First, Nicorandil pharmacologically dilates the coronary artery microvessels with a diameter of < 100 μm, increases coronary blood flow and decreases the heart preload [33]. Second, Nicorandil has myocardial protection in acute ischemic preconditioning, which exert effect of prevention of reperfusion injury as well as protection of myocardium from ischemic injury via ATP-sensitive K+ channels. In ischemic myocardium, intracellular ATP depletion can activate the K-ATP channels, leading to increased outflow of potassium, thereby the duration of the action potential shortens and the amount of calcium that flows into the myocytes reduces. Inhibiting and reducing the role of calcium in cardiomyocytes is thought to be a mechanism of cardioprotective effects on ischemic heart, and Nicorandil contributes to this effect as K-ATP channel opener. Since microvascular obstruction after myocardial infarction was associated with left ventricular remodelling, Nicorandil could improve left ventricular function for inhibiting the progression of microvascular damage and reperfusion arrhythmia. A previous study reported that intravenous infusion of Nicorandil in acute MI reduced myocardial injury and improved cardiac function, as shown by echocardiographic regional wall motion scoring and quantitative thallium single-photon emission computerized tomography analysis, respectively [34]. However, several studies [14, 21, 22] showed that Nicorandil did not significantly improve LVEF, which may be related to shorter follow-up time and baseline differences in the study population. In conclusion, LVEF is one of the most important factors affecting the prognosis of patients harboring coronary artery disease, and its improvement may lead to a decrease in the incidence of adverse cardiac events.

In addition, oral administration of Nicorandil can positively affect the activity of the cardiac sympathetic nerve [35], long-term administration of Nicorandil has good compliance with less adverse reactions [36]. Nicorandil has antihypertensive effects. These are also the reasons why Nicorandil could improve the prognosis of patients who are diagnosed with coronary artery disease.

There were several limitations in current analysis that should be acknowledged. First, there were only few eligible trials in some subgroups of meta-analyses, given the small and single-center nature of the current 14 RCTs with various quality, and only 2 RCTs [13, 18] were multicenter, large-scale clinical trials. Only 3 studies took the allocation of double-blind and hidden, the rest of the allocation of blind or hidden cases were unclear; only 7 studies of random grouping method were correct, with the rest insufficient. Second, there was a baseline inconsistency in the study, i.e. inconsistent intervention measures, including coronary and intravenous administration, continuous intravenous administration, dose of Nicorandil and subsequent long-term oral administration. Based on the aggregated results of current study, Nicorandil efficacy and dose-dependent relationship were not significant, with only part of the collection of fixed doses of Nicorandil research, thereby the current dose-dependent analysis of the relationship was inconclusive. Relevant researches were needed for further improvements in terms of the following aspects: right random allocation and allocation of hidden programs, larger sample size, longer follow-ups to further observe the short-term as well as long-term effects, identify the role of different routes of administration and the efficacy on dose, more thorough and comprehensive assessment with regard to the efficacy as well as the adverse reactions of Nicorandil.

## Conclusions

Moderate evidence has been collected in this systematic review that intracoronary/intravenous injection or oral Nicorandil is currently an acceptable and effective adjunctive therapy in patients who suffer from coronary artery disease and undergo elective PCI. It appears to be associated with the suppression of myocardium injury, the improved left ventricular function, and reduced incidence of adverse cardiac events at long term. However, considering the poor quality of the papers evaluated, additional high-quality RCTs are in great demand to confirm the effect of Nicorandil therapy accompanied with PCI in treating patients who suffer from coronary artery disease.
